# Association of *DPP4* Gene Variants with Classic and DPP4 Inhibitor-Associated Bullous Pemphigoid

**DOI:** 10.3390/ijms262311698

**Published:** 2025-12-03

**Authors:** Charoula Achilla, Christina Foutsitzidou, Parthena Meltzanidou, Aikaterini Patsatsi, Elizabeth Lazaridou, Glykeria Tzatzagou, Alexandros Lambropoulos, Anthoula Chatzikyriakidou

**Affiliations:** 1 Laboratory of Medical Biology-Genetics, Medical School, Aristotle University, 54124 Thessaloniki, Greece; 2 Genetics Unit, 1st Department of Obstetrics and Gynecology, Papageorgiou General Hospital, Medical School, Aristotle University, 56403 Thessaloniki, Greece; 3 Pemphig Center, Papageorgiou General Hospital, Medical School, Aristotle University, 56403 Thessaloniki, Greece; 4 2nd Department of Dermatology, Papageorgiou General Hospital, Medical School, Aristotle University, 56403 Thessaloniki, Greece; 5 1st Department of Internal Medicine, Papageorgiou General Hospital, Medical School, Aristotle University, 56403 Thessaloniki, Greece

**Keywords:** bullous pemphigoid, dipeptidyl peptidase-4 inhibitor (DPP4i), DPP4i-associated bullous pemphigoid, gliptins, *DPP4* gene, variant

## Abstract

Bullous pemphigoid (BP), the most prevalent autoimmune blistering skin disorder, has been associated with dipeptidyl peptidase-4 inhibitor (DPP4i) treatment in type 2 diabetic patients. This study aimed to investigate the association of *DPP4* gene variants, rs3788979 and rs12617656, with classic BP (cBP)- and DPP4i-associated BP predisposition. Fifty-six (56) unrelated patients with cBP, 32 DPP4i-associated BP patients, 60 healthy controls, and 49 diabetic patients receiving DPP4i were included. Genotyping was performed using polymerase chain reaction-restriction fragment length polymorphism assay (PCR-RFLP). Statistical analyses were conducted using SPSS software. For rs3788979, the CT+TT genotypes were significantly associated with increased risk of DPP4i-associated BP compared with cBP [(Odds Ratio (OR) = 2.80, 95% Confidence Interval (CI) = 1.07–7.35; *p*-value = 0.034] and healthy controls (OR = 0.30, 95% CI = 0.13–0.86; *p*-value = 0.020). The T allele was also enriched in DPP4i-associated BP (OR = 2.57, 95% CI = 1.09–6.07; *p*-value = 0.027). Additionally, the TC genotype of rs12617656 (OR = 2.29, 95% CI = 1.04–5.03, *p*-value = 0.039) showed significant association with cBP susceptibility. These findings highlight *DPP4* variants as potential BP risk factors, supporting personalized risk assessment prior to initiating gliptin therapy. Large-scale studies are warranted to validate these associations.

## 1. Introduction

Bullous pemphigoid (BP) is a rare, chronic, autoimmune blistering disease that predominantly affects the elderly and represents the most common autoimmune bullous disorder. It is characterized by the presence of autoantibodies targeting structural components of the basement membrane zone, particularly BP180 (type XVII collagen) and BP230. These autoantibodies mediate dermoepidermal separation, ultimately leading to subepidermal blister formation [1]. The pathogenesis of BP is multifactorial, involving genetic predisposition, immune dysregulation, and environmental triggers [2]. Among genetic factors, HLA class II alleles, especially HLA-DQB1*0301, are the most consistently associated with increased BP risk. However, recent studies suggest that additional immune-related loci may also influence disease susceptibility, severity, and clinical phenotype [2,3].

BP can arise as a primary idiopathic disorder (classic BP, cBP) or in association with pharmacologic treatment using dipeptidyl-peptidase 4 (DPP4) inhibitors, commonly known as gliptins, used in the management of type 2 diabetes (DPP4i-associated BP) [4]. DPP4, also known as CD26, is a transmembrane glycoprotein expressed on various cell types, including keratinocytes and T lymphocytes. It is involved in multiple biological processes such as immune regulation, cytokine signaling, and glucose homeostasis [5]. Although the mechanisms by which DPP4 inhibition contributes to BP pathogenesis remain unclear, it has been hypothesized that gliptins may alter the antigenic integrity of the epidermal basement membrane, thereby promoting autoimmunity in genetically susceptible individuals [6].

Given DPP4’s central role in immune modulation, genetic variation in the *DPP4* gene may influence susceptibility to BP. The human *DPP4* gene is located on chromosome 2, spans 70 kb, and consists of 26 exons [7]. Several *DPP4* variants have been associated with altered gene expression, protein levels, and immune pathways, as well as with various autoimmune and metabolic disorders [8,9,10,11,12,13,14]. However, despite the clinical association between DPP4 inhibition and BP onset, the specific contribution of *DPP4* genetic variants to BP risk has not been fully elucidated. This study aims to investigate the association of *DPP4* rs3788979 (C>T) and rs12617656 (T>C) gene variants to both cBP and DPP4i-asso-ciated BP predisposition, offering new insight into the genetic factors underlying disease risk. These variants were selected because previous studies linked rs3788979 to altered DPP4 expression and serum DPP4 levels [13], and rs12617656 to autoimmune and metabolic disease susceptibility [8,10].

## 2. Results

Genotype distributions of *DPP4* rs3788979 and rs12617656 gene variants were consistent with Hardy–Weinberg equilibrium (HWE) across all studied groups (Table 1).

### 2.1. rs3788979 Variant

Statistically significant differences in genotype and allele frequencies of rs3788979 were observed in the DPP4i-associated BP patients when compared to all other groups. Specifically, the T allele, as well as the CT and TT genotypes, were significantly more frequent in DPP4i-associated BP patients compared to healthy controls, cBP patients, and diabetic patients on gliptins (DPG) (Table 2). In contrast, no significant differences in rs3788979 genotype or allele frequencies were observed between cBP patients and healthy controls, suggesting a variant-specific association with the gliptin-exposed BP phenotype.

### 2.2. rs12617656 Variant

For rs12617656, the TC genotype was significantly associated with increased risk of cBP risk under the heterozygous model and a borderline statistical significance association observed under the dominant model (Table 2), indicating a potential contributory role of this genotype in cBP susceptibility. No significant associations were detected in the DPP4i-BP group or other control groups.

### 2.3. Linkage Disequilibrium (LD) Analysis

LD analysis revealed strong LD between rs3788979 and rs12617656 in the healthy control, cBP, and DPG groups (Table 3). In these groups, r^2^ values were consistently low, and D′ values were moderate to high. This LD pattern, as previously described in the literature [15], is characteristic of substantial allele frequency differences despite non-perfect correlation. In contrast, in the DPP4i-BP group, LD analysis showed lower *D′* value of 0.44 and *r*^2^ value of 0.09 indicating weaker LD between the two *DPP4* variants in this group.

### 2.4. Haplotype Analysis

To assess the combined effects of both *DPP4* variants, haplotype analysis was performed, identifying four main haplotypes (Table 3). Notably, the TT haplotype (carrying the T alleles at both rs3788979 and rs12617656) was significantly more frequent in DPP4i-BP patients compared with healthy controls [Odds Ratio (OR) = 10.084, 95% Confidence Interval (CI) = 1.151–88.292; *p*-value = 0.019], and DPG group (OR = 8.22, 95% CI = 0.937–72.091; *p*-value = 0.035]. These findings suggest that the TT haplotype may confer increased susceptibility to DPP4i-associated BP.

## 3. Discussion

The *DPP4* gene encodes a multifunctional enzyme involved in glucose metabolism, immune regulation, and inflammatory signaling. It is best known as the pharmacological target of gliptins, a widely used class of antidiabetic agents in type 2 diabetes mellitus [5]. A growing body of evidence has linked DPP4 inhibitor therapy with an increased risk of BP, the most common autoimmune blistering disorder in the elderly [4,6,16]. Genetic variations in the *DPP4* gene may modulate this risk by influencing enzyme levels or activity [13,17].

In this study, we investigated the potential role of two *DPP4* genetic variants, rs3788979 and rs12617656, previously implicated in cardiovascular, metabolic, and autoimmune diseases [8,9,10,18,19], in the context of both classic and DPP4i-associated BP.

Our findings revealed that the T allele, along with the CT and TT genotypes of rs3788979, was significantly associated with an increased risk of DPP4i-associated BP. Prior studies have reported that the T allele is associated with reduced *DPP4* gene expression and lower serum protein levels [13]. Given that low serum DPP4 levels have been linked to increased susceptibility of autoimmune conditions [20,21,22], we propose that carriers of the T allele may already have diminished enzymatic activity. When combined with the pharmacologic inhibition of DPP4 by gliptins, this genetically predisposed reduction could further impair DPP4 function. The resulting loss of immune regulatory activity, particularly in T-cell modulation and cytokine balance [23], may contribute to the breakdown of immune tolerance and the onset of autoimmunity, manifesting as BP.

DPP4 exists in both membrane-bound and soluble isoforms, each with distinct immunomodulatory functions [24]. While a previous study has shown increased membrane-bound DPP4 expression in BP skin lesions [25], this likely reflects local inflammatory upregulation. Conversely, carriers of the rs3788979 T allele may exhibit systematically reduced levels of soluble DPP4 predisposing them to immune dysregulation. This dual pattern is also observed in psoriasis, where serum DPP4 levels are decreased [26,27], whereas skin expression is elevated [28], suggesting that systemic and tissue-specific DPP4 dynamics may diverge in inflammatory skin diseases.

Regarding rs12617656, we observed that the TC genotype was significantly more frequent among patients with cBP, suggesting a possible role in cBP susceptibility. Although no functional studies have yet explored the effect of this variant on DPP4 expression, a recent genome-wide association study (GWAS) in the Chinese Han population reported a link between rs12617656 and rheumatoid arthritis, further implicating it in immune-mediated diseases [8]. Another study also associated this variant with an increased risk of type 2 diabetes [10]. These findings highlight the need for further research into how rs12617656 influences DPP4 isoforms and contributes to both metabolic and autoimmune pathophysiology.

Our haplotype analysis demonstrated that the TT haplotype (comprising the T alleles of both rs3788979 and rs12617656) was significantly enriched in DPP4i-associated BP patients compared to both healthy controls and the DPG control group. However, this association appears to be preliminary driven by rs3788979, reinforcing its role as the main genetic contributor to DPP4i-BP. By contrast, rs12617656 seems to exert a weaker, but possibly independent influence on cBP susceptibility.

It is also important to note that gliptin exposure was relatively homogeneous in our cohort. In the DPP4i-BP group, vildagliptin accounted for half of all exposures, and the DGP control group was uniformly treated with vildagliptin, the DPP4 inhibitor most strongly associated with BP [29,30]. This similarity in drug type minimizes confounding and supports the interpretation that genetic predisposition—rather than variation in gliptin therapy—is the primary factor underlying the increased risk of DPP4i-associated BP observed in carriers of the rs3788979 T allele.

To our knowledge, this is the first study to investigate the association of *DPP4* genetic variants in the context of BP susceptibility. Our results indicate that the rs3788979 T allele and its associated genotypes (CT and TT) are linked to increased DPP4i-associated BP, likely through effects on soluble DPP4 levels and immune function. Additionally, the rs12617656 TC genotype may primary and modestly increase susceptibility to cBP. Together, these findings offer new insights into the genetic underpinnings of BP and support the integration of genetic screening into clinical decision-making to optimize treatment strategies in patients receiving DPP4 inhibitors.

A similar pattern is seen in the *Thiopurine Methyltransferase (TPMT)* gene, where different variants influence disease risk through distinct mechanisms. For example, rs1142345 is a key determinant of thiopurine toxicity, whereas rs1800460 has been linked to inherent leukemia susceptibility independent of drug exposure [31,32,33,34,35]. This illustrates how variants within the same gene may contribute to risk either pharmacogenetically or intrinsically, underscoring the importance of personalized medicine.

To date, only a limited number of studies have examined the genetic architecture of DPP4i-associated BP, primarily implicating alleles within the HLA region [36,37,38]. Our study extends this understanding by highlighting a potential non-HLA genetic factor, thereby reinforcing the concept that both genetic predisposition and pharmacological exposure act synergistically in DPP4i-BP pathogenesis. In the context of personalized medicine and pharmacogenetics, the identification of high-risk *DPP4* genotypes could enable more tailored therapeutic approaches for patients with type 2 diabetes mellitus, helping clinicians balance glycemic control with autoimmune disease risk.

Nonetheless, our study has limitations. The modest sample size and the low frequency of certain genotypes may lead to unstable OR estimates; for example, the TT genotype of rs3788979 was observed in only one DPP4i-BP patient and one control, resulting in wide confidence intervals and necessitating cautious interpretation. Given the exploratory nature of the study and the relatively small cohort, we did not apply a formal correction for multiple comparisons. This approach aligns with prior pilot or small-scale investigations, including both hypothesis-generating and validation studies [39], and reflects concerns that overly conservative thresholds can reduce power in exploratory genetic research [40]. Additionally, data on the duration of DPP4 inhibitor therapy were not consistently documented and therefore could not be analyzed, which may limit our ability to fully evaluate exposure–outcome relationships. Therefore, our findings require validation in larger, multi-center cohorts, and functional studies are needed to determine how these variants affect *DPP4* gene expression or enzymatic activity in relevant tissues, further elucidating the molecular mechanisms linking DPP4 function to BP pathogenesis.

## 4. Materials and Methods

The study population included patients with a confirmed diagnosis of BP, stratified into two groups based on gliptin exposure: 56 unrelated non–gliptin-treated patients with cBP (26 males, 31 females; mean age 78 ± 11 years) and 32 unrelated diabetic patients with DPP4i-associated BP (19 males, 13 females; mean age 80 ± 7 years). Patients were enrolled consecutively from the Pemphig Center and 2nd Department of Dermatology at Papageorgiou University Hospital.

Diagnosis of BP was established according to internationally accepted criteria [41], based on histopathological findings of tense subepidermal blistering, and confirmed by direct immunofluorescence (DIF) demonstrating linear IgG and/or C3 deposition at the dermal–epidermal junction. Additionally, serological testing for circulating BP180 and BP230 autoantibodies was performed using enzyme-linked immunosorbent assays (ELISA) (Euroimmun, Lübeck, Germany), with a diagnostic threshold of 20 RU/mL applied per manufacturer’s instructions. The mean serum antibody levels in BP patients were 140.13 ± 109.6 RU/mL for BP180 and 133.53 ± 121.68 RU/mL for BP230. ELISAs were performed using the Dynex Technologies ELISA Testing System Dynex Technologies (Chantilly, VA, USA), while DIF was conducted on perilesional biopsies according to established protocols [42].

The study additionally included two control groups: 60 healthy controls (29 males, 31 females; mean age 77 ± 12 years), and 49 DPG with no history or diagnosis of BP. Healthy controls were ethnically matched individuals recruited from routine outpatient clinics of Papageorgiou General Hospital, meeting predefined criteria of no personal history of autoimmune or chronic infectious disease. Type 2 diabetes mellitus was defined according to the diagnostic criteria of the American Diabetes Association (ADA), and all diabetes controls fulfilling these criteria were recruited from the 1st Department of Internal Medicine at Papageorgiou General Hospital [43].

In the DPP4i-BP group, the specific DPP4 inhibitors administered were vildagliptin in 16 of 32 patients (50%), alogliptin in 2 (6%), linagliptin in 5 (16%), and sitagliptin in 1 (3%), while medication data were unavailable for 8 patients (25%). In contrast, all participants in the DGP group were treated with vildagliptin, selected due to its previously reported higher association with BP [29,30]. Treatment duration was available only for the DGP group (mean 5 ± 1.8 years), as longitudinal diabetes therapy data are routinely documented in Internal Medicine, whereas such information was not consistently available for DPP4i-BP patients presenting to Dermatology at the time of BP onset.

Sample size estimations were conducted to ensure a 80% confidence level and a significance probability of 0.05, in accordance with recognized recommendations for preliminary studies [44].

Genomic DNA was extracted from peripheral blood lymphocytes using the PureLink Genomic DNA Kit (Invitrogen, Karlsruhe, Germany), according to the manufacturer’s instructions. Genotyping of the *DPP4* variants rs3788979 (intron) and rs12617656 (intron) was conducted via polymerase chain reaction–restriction fragment length polymorphism (PCR-RFLP) analysis. Each PCR reaction (25 µL final volume) contained 100 ng of genomic DNA, 2 µM of each primer, 2 units of OneTaq DNA Polymerase (New England Biolabs, Ipswich, MA, USA), 1× reaction buffer, 0.2 µM dNTP mix (New England Biolabs, Ipswich, MA, USA), and nuclease-free water. Primer pairs specific to each target region were designed using Primer 3.0 software [45,46], with sequences listed in Table 4. Thermal cycling conditions were as follows: initial denaturation at 95 °C for 5 min; 33 cycles of denaturation at 95 °C for 30 s, annealing at 56 °C for 30 s, and extension at 72 °C for 1.5 min; followed by a final extension at 72 °C for 10 min. PCR products were digested with the appropriate restriction endonucleases (New England Biolabs, Ipswich, MA, USA; Table 4), and digested products were separated by electrophoresis on 3% agarose gels. To ensure accuracy, all genotyping assays were performed in duplicate.

Statistical analyses were performed using SPSS version 28.0 (IBM Corp., Armonk, NY, USA). HWE for each variant, rs3788979 and rs12617656, was assessed in all studied groups using Pearson’s chi-square test. Differences in genotype and allele frequencies bet-ween groups were evaluated using Pearson’s chi-square test or Fisher’s exact test (for expected cell counts < 5), under seven genetic association models: additive, dominant, recessive, homozygous, heterozygous, overdominant, allelic. Odds ratio (OR) with 95% Confidence Intervals (CI) were calculated. Where contingency tables containing zero values, the Haldane–Anscombe correction was applied [47,48].

LD and haplotype frequencies between rs3788979 and rs12617656 were estimated using SHEsisPlus software (http://shesisplus.bio-x.cn/SHEsis.html, assessed on 12 September 2025) [49]. A *p*-value ≤ 0.05 was considered statistically significant in all analyses.

## Figures and Tables

**Table 1:** Genotype distributions of *DPP4* gene rs3788979 and rs12617656 variants in patients with classic Bullous Pemphigoid (cBP), dipeptidyl peptidase-4 inhibitor (DPP4i)-associated BP, healthy controls and, diabetic patients on gliptins (DPG).

Genotypes	Genotype Frequencies, *n* (%)			
	cBP Patients (*n* = 56)	DPP4i-Associated BP Patients (*n* = 32)	Healthy Controls (*n* = 60)	DPG (*n* = 49)
**rs3788979**				
CC	45 (80.357)	19 (59.375)	49 (81.667)	41 (83.673)
CT	11 (19.643)	12 (37.5)	10 (16.667)	8 (16.327)
TT	0	1 (3.125)	1 (1.667)	0
HWE ^1^ (*p*-Value)	0.415	0.583	0.566	0.534
**rs12617656**				
TT	20 (35.714)	13 (40.625)	32 (53.333)	20 (40.816)
TC	30 (53.571)	15 (46.875)	21 (35)	21 (42.857)
CC	6 (10.714)	4 (12.5)	7 (11.667)	8 (16.327)
HWE ^1^ (*p*-Value)	0.234	0.919	0.182	0.537

^1^ HWE: Hardy–Weinberg Equilibrium.

**Table 2:** Significant associations of *DPP4* gene rs3788979 and rs12617656 variants with classic Bullous Pemphigoid (cBP) and dipeptidyl peptidase-4 inhibitor (DPP4i)-associated BP susceptibility under different genetic models.

Compared Groups	Statistical Model	OR (95%CI) ^1^	*p*-Value
**rs3788979**			
cBP vs. DPP4i-associated BP	Additive (CC vs. CT vs. TT)		0.04
	Heterozygous (CC vs. CT)	2.584 (0.971–6.872)	0.053
	Dominant (CC vs. CT)	2.799 (1.066–7.351)	0.034
	Allelic (C vs. T)	2.571 (1.089–6.072)	0.027
DPP4i-associated BP vs. Healthy Controls	Additive (CC vs. CT vs. TT)		0.04
	Heterozygous (CC vs. CT)	0.323 (0.12–0.872)	0.022
	Dominant (CC vs. CT)	0.298 (0.125–0.859)	0.02
	Overdominant (CT vs. CC+TT)	3 (1.119–8.046)	0.026
	Allelic (C vs. T)	0.397 (0.171–0.92)	0.028
DPP4i-associated BP vs. Diabetic patients on gliptins (DPG)	Additive (CC vs. CT vs. TT)		0.013
	Heterozygous (CC vs. CT)	0.31 (0.108–0.88)	0.024
	Dominant (CC vs. CT)	0.285 (0.101–0.803)	0.015
	Overdominant (CT vs. CC+TT)	3.075 (1.085–8.718)	0.031
	Allelic (C vs. T)	0.318 (0.125–0.809)	0.013
**rs12617656**			
cBP vs. Healthy Controls	Heterozygous (TC vs. TT)	2.286 (1.038–5.033)	0.039
	Dominant (TC+CC vs. TT)	2.057 (0.976–4.336)	0.056

^1^ OR: Odds Ratio; 95% CI: 95% Confidence Interval.

**Table 3:** Haplotype frequencies and linkage disequilibrium between *DPP4* gene rs3788979 and rs12617656 variants across the studied groups.

rs3788979/rs12617656 Haplotypes	Haplotype Frequencies, *n* (%)			
	Classic Bullous Pemphigoid Patients (cBP)	Dipeptidyl Peptidase-4 (DPP4i)-Associated BP Patients	Healthy Controls	Diabetic Patients on Gliptins (DPG)
TC	70 (62.5)	36 (56.25)	84 (70)	60 (61.225)
CC	31 (27.679)	14 (21.875)	24 (20)	30 (30.612)
CT	11 (9.821)	9 (14.063)	11 (9.167)	7 (7.143)
TT	0	5 (7.813)	1 (0.833)	1 (1.02)
Linkage disequilibrium				
D′	1	0.44	0.88	0.79
r^2^	0.18	0.09	0.21	0.09

**Table 4:** Primer sequences and restriction enzymes used in polymerase chain reaction–restriction fragment length polymorphism (PCR –RFLP) analysis for genotyping of *DPP4* rs3788979 and rs12617656 variants.

*DPP4* Gene Variant	Primer Sequences	Amplicon Length (bp: Base Pairs)	Restriction Enzyme	Restriction Digestion Pattern
**rs3788979**	Forward: 5′ GCCCAGCAAATCCAGGGTAA 3′ Reverse: 5′ GGGATTCCCACCCCTGATCT 3′	266 bp	HaeIII	Τ allele: 266 bp C allele: 81 bp, 185 bp
**rs12617656**	Forward: 5′ ACAACAGCTCTAGCCATTCCT 3′ Reverse: 5′ AGACACTGCTCTCCTGTTCA 3′	303 bp	MluCI	C allele: 40 bp, 43 bp, 220 bp Τ allele: 40 bp, 43 bp, 93 bp, 127 bp

## Data Availability

The original contributions presented in this study are included in the article. Further inquiries can be directed to the corresponding author.

## Abbreviations

The following abbreviations are used in this manuscript:

BP	Bullous Pemphigoid
cBP	Classic Bullous Pemphigoid
CI	Confidence Interval
DIF	Direct Immunofluorescence
DPG	Diabetic Patients on Gliptins
DPP4	Dipeptidyl-Peptidase 4
ELISA	Enzyme-Linked Immunosorbent Assays
HWE	Hardy–Weinberg Equilibrium`
LD	Linkage Disequilibrium
OR	Odds Ratio
PCR-RFLP	Polymerase Chain Reaction–Restriction Fragment Length Polymorphism
TPMT	Thiopurine Methyltransferase
